# Prealbumin/CRP Based Prognostic Score, a New Tool for Predicting Metastasis in Patients with Inoperable Gastric Cancer

**DOI:** 10.1155/2016/4686189

**Published:** 2016-01-20

**Authors:** Ali Esfahani, Nima Makhdami, Elnaz Faramarzi, Mohammad Asghari Jafarabadi, Alireza Ostadrahimi, Mousa Ghayour Nahand, Zohreh Ghoreishi

**Affiliations:** ^1^Hematology and Oncology Research Center, Tabriz University of Medical Sciences, Tabriz 5166614711, Iran; ^2^School of Medicine, Tabriz University of Medical Sciences, Tabriz 5166614711, Iran; ^3^Liver and Gastrointestinal Disease Research Center, Tabriz University of Medical Sciences, Tabriz 5166614711, Iran; ^4^Tabriz Health Services Management Research Center and Department of Statistics and Epidemiology, Faculty of Health and Nutrition, Tabriz University of Medical Sciences, Tabriz 5166614711, Iran; ^5^Nutrition Research Center, Tabriz University of Medical Sciences, Tabriz 5166614711, Iran; ^6^Common Diseases Risk Factors Management Institute, Tabriz University of Medical Sciences, Tabriz 5166614711, Iran

## Abstract

*Background.* There is a considerable dissimilarity in the survival duration of the patients with gastric cancer. We aimed to assess the systemic inflammatory response (SIR) and nutritional status of these patients before the commencement of chemotherapy to find the appropriate prognostic factors and define a new score for predicting metastasis.* Methods.* SIR was assessed using Glasgow Prognostic Score (GPS). Then a score was defined as prealbumin/CRP based prognostic score (PCPS) to be compared with GPS for predicting metastasis and nutritional status.* Results.* 71 patients with gastric cancer were recruited in the study. 87% of patients had malnutrition. There was a statistical difference between those with metastatic (*n* = 43) and those with nonmetastatic (*n* = 28) gastric cancer according to levels of prealbumin and CRP; however they were not different regarding patient generated subjective global assessment (PG-SGA) and GPS. The best cut-off value for prealbumin was determined at 0.20 mg/dL and PCPS could predict metastasis with 76.5% sensitivity, 63.6% specificity, and 71.4% accuracy. Metastatic and nonmetastatic gastric cancer patients were different in terms of PCPS (*P* = 0.005).* Conclusion.* PCPS has been suggested for predicting metastasis in patients with gastric cancer. Future studies with larger sample size have been warranted.

## 1. Introduction

The majority of patients with gastric cancer have a poor overall survival. Therefore, finding the appropriate prognostic factors will help to improve clinical approach on patients that will lead to accurate decision making and planning for supportive care. This will possibly increase the rate of survival [1].

Weight loss and performance status are usually used to predict survival and treatment outcomes in patients with inoperable gastric adenocarcinoma [2], but the degree to which they are associated with poor prognosis is not well defined and performance status does not provide an objective measurement [3, 4]. Studies have shown that the presence of malnutrition and a systematic inflammatory response cause a short survival, reduced response rate, and higher risk for treatment-induced complications in patients with malignancy [5, 6].

Recently, the host nutritional and immune status have been evaluated by the Glasgow Prognostic Score (GPS), which is a combination of serum C-reactive protein (CRP) as an index for systemic inflammatory response and an important factor for the development and progression of neoplasms [7] along with serum albumin which has been proposed as a prognostic factor in a variety of cancers. GPS has prognostic importance independent of tumor stage in number of malignancies including gastrointestinal cancer [4, 8–10].

On the other hand, prealbumin is a remarkable prognostic factor for treatment outcomes and/or nutritional status of colon [11], esophagus [12], ovarian [13], and lung cancers [14, 15]. Recently, we studied the nutritional status of patients with acute lymphoblastic leukemia (ALL) and acute myeloid leukemia (AML) during induction chemotherapy and its effects on chemotherapy-related complications in which prealbumin was found as the common biomarker for better treatment outcomes in both groups of patients with acute leukemia [16]. Here, systemic inflammatory response of the patients with inoperable gastric adenocarcinoma (IGA) was investigated by GPS, while their nutritional status was assessed using patient generated subjective global assessment (PG-SGA) as well as the serum levels of albumin, prealbumin, transferrin, CRP, and total lymphocyte count (TLC). Then a new prognostic score, prealbumin/CRP based prognostic score (PCPS), was introduced for predicting metastasis in this group of patients based on serum prealbumin and CRP and compared with PG-SGA and GPS.

## 2. Materials and Methods

### 2.1. Study Population

A convenient sample of 71 patients with inoperable gastric adenocarcinoma was recruited in this prospective study before the onset of chemotherapy between February 2013 and March 2014 (Table 1). The Human Ethics Committee of Tabriz University of Medical Sciences approved the study and written informed consent was obtained from all the patients before the commencement of the study. Patients with history of other malignancies, autoimmune disease, chronic renal or hepatic disease, diabetes, and thyroid disorders and those who were taking anti-inflammatory drugs were excluded from the study. The Tumor-Node-Metastasis (TNM) classification of malignant tumors was used for staging the tumors. All patients received the same chemotherapy regimen as described below.

The chemotherapy regimen was as follows: docetaxel, 75 mg/m^2^ i.v. (one-hour infusion) on day 1; cisplatin, 75 mg/m^2^ i.v. (one-hour infusion) on day 1; and 5-fluorouracil (5-FU) i.v. (continuous infusion) on days 1–5, to be repeated every 3 weeks for 6 cycles.

### 2.2. Biochemical Analyses

Venus blood samples were taken after an overnight fasting and the serum was separated and stored at −70°C for future analysis. Hitachi 917 automated equipment was used for measuring albumin concentration and serum CRP, prealbumin, and transferrin were analyzed using the Minineph Human kits (Birmingham, UK).

### 2.3. Immunological Analyses

The systemic inflammatory response was measured using a combination of serum C-reactive protein and albumin as follows: patients with C-reactive protein ≤10 mg/L and albumin ≥3.5 mg/dL were allocated a score of 0; patients with one of these parameters abnormalities were allocated a score of 1; and those with both abnormalities, C-reactive protein >10 mg/L and albumin <3.5 g/dL, were allocated a score of two (Table 2). Then a new score was defined and called the prealbumin/CRP based prognostic score (PCPS) and it was compared with conventional GPS for evaluating the inflammatory status of patients and predicting metastasis. The PCPS was constructed using prealbumin and C-reactive protein with the same cut-off value for CRP and 0.20 mg/dL for prealbumin. Similar categorization was used for allocating scores of 0, 1, and 2 to the patients.

### 2.4. Nutritional Assessment

BMI was computed as weight (kg)/height (m^2^). The scored PG-SGA was completed by all the patients with the help of a trained oncology nurse. PG-SGA consists of the history of weight changes, food intakes, and the contributing factors, activities, physical examination, and the metabolic stress which affects the nutritional requirements. Based on scored PG-SGA, patients were scored at 0-1 (with no need of nutritional intervention) and there was a progressive need for nutritional support, so that those who scored ≥9 required immediate symptom management and/or nutritional support. PG-SGA also provided a categorical assessment as PG-SGA A (well-nourished), PG-SGA B (moderate malnutrition), and PG-SGA C (severe malnutrition) [5].

### 2.5. Statistical Analyses

Quantitative variables were presented as mean (standard deviation [SD]) or median (percentile 25–percentile 75) based on the normality of the distribution, and qualitative variables were reported as frequency (%). The best cut-off point value for prealbumin was determined using receiver operating characteristic (ROC) analysis, considering the optimal sensitivity and specificity to calculate the Youden Index ((specificity + sensitivity) − 1). The likelihood ratios (LRs) and area under curve (AUC) were shown as a measure of metastasis prediction adequacy using prealbumin concentration. Then regression tree analysis was used to measure sensitivity, specificity, and accuracy of the new score, PCPS, for prediction of metastasis in IGA patients.

The association between categorized variables was examined using Chi-square test or Fisher exact test. Independent-samples Kruskal-Wallis test was used to determine the association between scored PG-SGA and categorized variables.

The significance level was considered 0.05 by doing a two-tailed analysis. SPSS software (SPSS Inc., Chicago, IL) was used for performing statistical analyses.

## 3. Results

**Figure 1:**
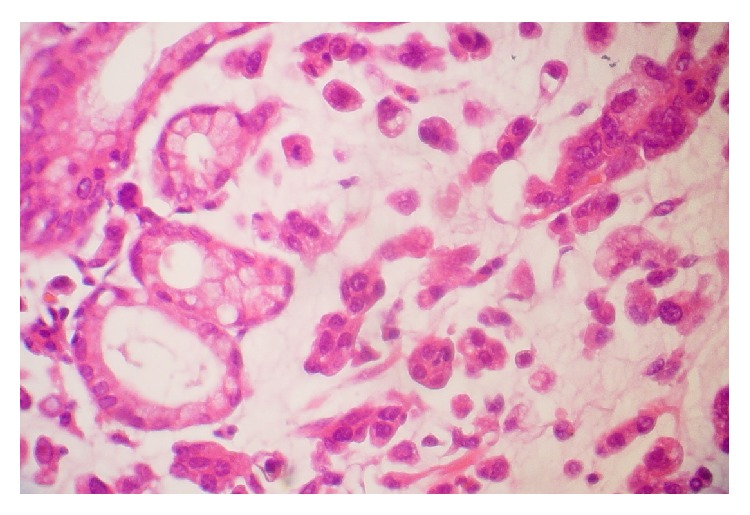
Microscopic scheme of metastatic diffuse type gastric cancer.

**Figure 2:**
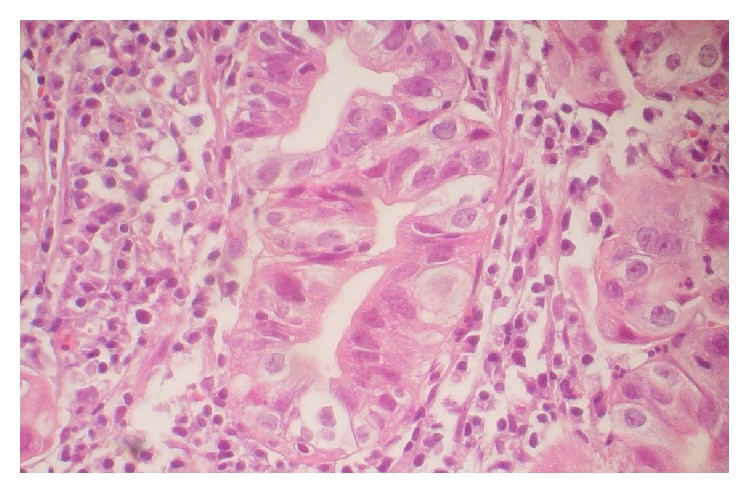
Microscopic scheme of nonmetastatic intestinal type gastric cancer.

General characteristics of patients are shown in Table 1. Seventy-nine percent (*n* = 56) of the patients were male and 21% (*n* = 15) were female with an average of 62.13 ± 14.39 and 21.08 ± 3.99 for age and BMI, respectively. Twenty-eight patients (39%) had locally advanced unresectable gastric cancer (stage 3) (Figure 1) and 43 patients (61%) had distance metastasis (stage 4) (Figure 2). According to categorized PG-SGA, 13% of patients were well-nourished (PG-SGA A), while 49% of them were moderately (PG-SGA B) and 38% severely (PG-SGA C) malnourished. Considering PG-SGA A as an index for well-nourished category and PG-SGA B and PG-SGA C for some degrees of malnutrition, 87% of patients suffered from malnutrition before the beginning of chemotherapy. The mean score for PG-SGA was 16.07 ± 5.02, an indicative of need for immediate nutritional support.

Then metastatic and nonmetastatic patients were compared in terms of BMI, scored PG-SGA, visceral proteins, CRP, and TLC. There was a statistical difference between them according to prealbumin and CRP; *P* = 0.012 and *P* = 0.004, respectively (Table 3).

The best cut-off value for prealbumin was determined at 0.20 mg/dL for differentiating metastatic from nonmetastatic status using ROC analysis (Table 4). Using regression tree analysis, PCPS could predict metastasis with 76.5% sensitivity, 63.6% specificity, and 71.4% accuracy (considering scores 0 and 1 in one category and score 2 in the second category).

There was no statistical association between GPS, PCPS, and categorized PG-SGA, although 72% of the patients with severe malnutrition had a score of 2 for PCPS (Table 5). Moreover, the distribution of scored PG-SGA was the same across the categories of GPS and PCPS (*P* = 0.527 and *P* = 0.334).

There was a statistical difference in PCPS score between metastatic and nonmetastatic gastric cancer patients (*P* = 0.009), while they were not different in terms of GPS (Table 6). Moreover, there was no statistical association between anatomic location and site of gastric adenocarcinoma and PCPS (*P* = 0.701 and *P* = 0.956, resp.).

## 4. Discussion

Malnutrition and systemic inflammatory response are common in patients with cancer and they both have significant impact on patients' quality of life, treatment outcomes, prognosis, and survival [4, 5, 15, 17]. In this study, the nutritional and inflammatory status of patients with inoperable gastric adenocarcinoma were assessed using known scores of PG-SGA, a valid tool for nutritional assessment of patients with cancer and GPS, a score for measuring systemic inflammation, and an independent prognostic score in different kinds of malignancies including gastrointestinal cancer [4, 8–10]. Eighty-seven percent of patients had some degrees of malnutrition and 65% of them had GPS scores of 1 or 2, but there was no significant difference between patients with metastatic and nonmetastatic gastric cancer in terms of PG-SGA or GPS.

Comparing patients with metastatic and nonmetastatic gastric cancer, it was found that they were different statistically according to prealbumin and CRP and not albumin. The results of this study confirm the findings of previous studies that showed that baseline levels of prealbumin had a significant correlation to overall survival in patients with advanced colorectal cancer [11] and esophageal cancer [12]. In a study on patients with non-small cell lung cancer [15] and epithelial ovarian carcinoma [18] prechemotherapy concentrations of prealbumin were associated with response to treatment and outcomes. Moreover, Ho et al. found that low level of prealbumin was an independent prognostic factor for overall survival in cancer patients and its assessment has been suggested to be considered as a part of palliative care setting [18].

Notably, Inoue et al. investigated the association between the serum levels of rapid turnover proteins (RTPs) and the prognosis in patients with advanced cancer receiving total parenteral nutrition. They found that there was a significant association between RTPs' concentration and survival in cancer patients and among RTPs and prealbumin had the most correct prognosis rate with 91.9% compared to transferrin and retinol binding protein [19]. The short prealbumin half-life of ≈2 days in the blood circulation makes it a more sensitive biomarker for the assessment of nutritional state compared to albumin with much longer half-life [20, 21]. It should be mentioned that we studied the nutritional status of patients with ALL and AML during induction chemotherapy and its impact on chemotherapy-related complications in which prealbumin was the common biomarker for better treatment outcomes in both groups of patients with acute leukemia [16].

On the other hand, CRP is an indicator of systemic inflammatory response and studies have shown the independent prognostic value of elevated serum levels of CRP in solid tumors including gastroesophageal cancer [6]. Given the evidences, this study designed a new prognostic score based on prechemotherapy concentrations of prealbumin and CRP, after determining the best cut-off point value of prealbumin by ROC analysis at 0.20 mg/dL for differentiating metastatic from nonmetastatic status named PCPS. This score could predict metastasis with 76.5% sensitivity, 63.6% specificity, and 71.4% accuracy. Noteworthy, the patients with metastatic and nonmetastatic gastric cancer were significantly different according to PCPS unlike the conventional score of GPS. However, there was no significant association between GPS, PCPS, and categorized PG-SGA which may be due to the relatively small sample size and the fact that 87% of the patients (metastatic and nonmetastatic) already had malnutrition (before the onset of chemotherapy). So PCPS could not be considered as a tool for assessment of nutritional status in patients with inoperable gastric adenocarcinoma.

The small sample size was one of the possible limitations, as it was a single center study and a large number of eligible patients declined inclusion as a result of their critical conditions. Moreover, there was no official registration system, so the patients could not be followed to determine the survival rate. However, this study presented a composite score of prealbumin and CRP suggesting that it may be a strong prognostic score in patients with inoperable gastric adenocarcinoma. Although it is early to propose the replacement of GPS with PCPS, assessment of the host inflammatory response and nutritional status with PCPS and evaluation of its association with response to treatment, prognosis, complications, and survival in patients with different kinds of cancer is warranted.

## Figures and Tables

**Table 1:** General characteristics of the patients and disease.

		Male	Female
Age (mean ± SD)	62.13 ± 14.39	63.43 ± 13.75	57.00 ± 16.24
Gender (*n*, %)			
Male	56 (79%)		
Female	15 (21%)		
Anatomic area (%)			
GEJ^*∗*^/proximal stomach	38 (54%)	29 (51%)	10 (64%)
Distal stomach	33 (46%)	27 (49%)	5 (36%)
Stage (%)			
3	28 (39%)	21 (37%)	6 (40%)
4	43 (61%)	35 (63%)	9 (60%)
BMI^#^ (mean ± SD)	21.08 ± 3.99		
Metastasis			
Metastatic	43 (61%)	21 (37%)	6 (40%)
Nonmetastatic^$^	28 (39%)	35 (63%)	9 (60%)
SGA A	13%	15%	9%
SGA B	49%	46%	58%
SGA C	38%	39%	33%
PG-SGA (mean ± SD)	16.07 ± 5.02	15.76 ± 5.17	16.92 ± 4.70

^*∗*^Gastroesophageal junction.

^*∗∗*^Type of gastric adenocarcinoma.

^#^Body mass index.

^$^Unresectable gastric cancer.

**Table 2:** Classification of prealbumin/CRP based prognostic score (PCPS).

Prealbumin (mg/dL)	CRP (mg/L)	PCPS
0.20<	<10	0
0.20<	10≤	1
<0.20	<10	1
<0.20	10≤	2

**Table 3:** Comparison between patients with metastatic and nonmetastatic inoperable gastric adenocarcinoma based on indicators of nutritional and inflammatory status.

	Metastatic	Nonmetastatic^$^	Mean difference (95% CI)	*P* value
BMI^*∗*^	21.94 ± 3.94	22.13 ± 4.11	0.18 (−2.43 to 2.81)	0.885
Albumin^*∗*^	3.57 ± 0.74	3.94 ± 0.68	0.36 (−0.30 to 0.76)	0.070
Prealbumin^*∗*^	0.14 ± 0.06	0.20 ± 0.10	0.06 (0.01 to 0.09)	**0.012**
Transferrin^*∗*^	218.48 ± 119.68	279.07 ± 135.75	60.59 (−5.75 to 126.94)	0.073
CRP^*∗∗*^	37.60 (15.59–85.16)	15.61 (5.52–30.01)		**0.004**
TLC^*∗*^	1.18 ± 0.49	1.27 ± 0.54	0.09 (−0.19 to 0.37)	0.516
PG-SGA^*∗∗*^	17 (13–20.50)	17 (11–19)	0.564	0.564

^*∗*^Mean ± SD, *P* value based on independent-samples *t*-test.

^*∗∗*^Median (percentiles 25–75), *P* value based on Mann-Whitney test (only *P* value was reported).

^$^Unresectable gastric cancer.

**Table 4:** Receiver operating characteristic (ROC) analysis and optimum cut-off point of prealbumin for predicting metastasis in patients with inoperable gastric adenocarcinoma.

	PA	AUC	SEN	SPE	PPV	NPV	LR^+^	LR^−^
mets	0.20	0.68	77.1%	52.2%	71.1%	60.0%	1.61	0.44
		(0.54–0.82)^*∗*^	(61.0–87.9)	(33.0–70.8)	(55.2–83.0)	(38.7–78.1)	(1.01–2.56)	(0.21–0.90)

Mets: metastasis; ^*∗*^95% confidence interval (CI); PA: prealbumin (mg/dL); AUC: area under the curve; SEN: sensitivity; SPE: specificity; LR^+^: positive likelihood ratio; LR^−^: negative likelihood ratio; NPV: negative predictive value; PPV: positive predictive value.

**Table 5:** The association between GPS, PCPS, and categorized PG-SGA.

	PG-SGA^*∗*^	PG-SGA	PG-SGA	*P* value^*∗∗*^
	A (%)	B (%)		
GPS^#^				
0	40	19	33	
1	20	52	25	
2	40	29	42	0.527
PCPS^$^				
0	40	14	14	
1	40	19	14	
2	20	67	72	0.334

^*∗*^Patient generated subjective global assessment.

^*∗∗*^ *P* value was calculated based on independent-samples Kruskal-Wallis test.

^#^Glasgow Prognostic Score.

^$^Prealbumin/CRP based prognostic score.

**Table 6:** The differences between metastatic and nonmetastatic patients with inoperable gastric adenocarcinoma in terms of PG-SGA, GPS, and PCPS.

	Metastatic	Nonmetastatic	*P* value^*∗∗*^
PG-SGA^*∗*^ (%)			
A	12	19	
B	48	44	
C	40	37	0.820
GPS^#^ (%)			
0	16	36	
1	40	41	
2	44	23	0.153
PCPS^$^ (%)			
0	9	27	
1	15	37	
2	76	36	**0.009**

^*∗*^Patient generated subjective global assessment.

^*∗∗*^ *P* value was calculated based on exact Chi-square test.

^#^Glasgow Prognostic Score.

^$^Prealbumin/CRP based prognostic score.

## Abbreviations

GPS: Glasgow Prognostic Score
CRP: C-reactive protein
ALL: Acute lymphoblastic leukemia
AML: Acute myeloid leukemia
PG-SGA: Patient generated subjective global assessment
TLC: Total lymphocyte count
PCPS: Prealbumin/CRP based prognostic score
RTPs: Rapid turnover proteins.
